# School closures and patterns of hospital admissions with stress-related presentations in secondary school aged adolescents

**DOI:** 10.1192/bjp.2022.113

**Published:** 2022-08-25

**Authors:** Ruth M Blackburn, Jacquie Phillips Owen, Johnny Downs, Ruth Gilbert

**Affiliations:** 1 UCL Institute of Health Informatics, London, UK; 2 Institute of Psychiatry, Psychology & Neuroscience, King’s College London, London, UK; 3 South London and Maudsley NHS Foundation Trust, London, UK; 4 UCL Great Ormond Street Institute of Child Health, London, UK

## Background

Significant declines in the mental health and wellbeing of children and adolescents have been reported during the coronavirus (COVID-19) pandemic, with the closure of schools and subsequent disruption to learning and assessment, loss of social contacts, routines and services accessed through schools playing a pivotal role [1].

Prior to the pandemic, hospital admissions relating to manifestations of stress in secondary school aged adolescents were positively correlated with term times [2]. Stress-related presentations are those relating to pain, mental health or psychosomatic symptoms. These may indicate emerging or relapsing mental illness [3] and the burden on schools and in health is significant, affecting 7.9% of girls and 4.1% of boys between the ages of 11 and 17 years, and accounting for over 30% of all emergency admissions for this age group [2]. Rates of stress-related presentations were highest in girls aged 14-15 years, and consistently higher in term times than holiday periods for children of secondary school age in 2014/15-2017/18 [2].

We aimed to investigate the impact of the pandemic and lockdown (including school closures) on rates of emergency hospital admissions with a stress-related presentation. Our study examined secondary school aged children to better characterise the relationships between schools and stress.

## Method

### Participants

We used Hospital Episodes Statistics (HES) data for all adolescents aged 11 to 17 who were admitted to an NHS hospital in England in the academic years (September 1^st^-August 31^st^) of 2018/19, 2019/20 and 2020/21. These years were selected to cover pre-pandemic and pandemic periods. We excluded records for adolescents of unknown sex or who were not resident in England.

### Measures of stress

HES data on hospital admissions captures data on up to 20 distinct diagnoses coded using the International Classification of Diseases (ICD) version 10. Stress-related presentations were defined as emergency hospital admissions with a primary diagnosis relating to mental health, pain without a medical cause, or potentially psychosomatic symptoms (e.g. fatigue), or where a diagnostic code indicating self-harm was recorded in any diagnostic position. Codes are outlined in Supplementary Tables 1 and 2 and were grouped into mutually exclusive categories relating to mental health/behavioural or pain/somatic presentations [2]. Admissions were excluded if these related to maternity care or where the adolescent died prior to discharge, because coding may differ relative to those discharged alive.

### Analysis

We estimated weekly rates of admissions with a stress-related presentation (per 100,000 girls or boys) using Office for National Statistics mid-year population estimates for England stratified into 1 year age bands. Weekly rates of stress-related presentations were plotted and visualised against school term times, which were defined from a sample of school timetables published online by local authorities in England. Incident rate ratios (IRRs) for term vs holiday time occurrence of stress-related presentations were estimated using separate negative binomial regression models that were age- and sex-specific, and adjusted for academic year (fitted as a categorical variable).

### Approvals

Approvals for the use of HES data were obtained from NHS Digital through the standard approval process. This study is exempt from NHS Research Ethics Committee approval and participant consent was not applicable because we analysed an existing dataset of deidentified data that was collected as part of routine care.

## Results

We identified a total of 662,000 emergency admissions for adolescents aged 11-17 years. Of these, 21,818 were excluded because they related to; exact duplicates (n=260), adolescents not residing in England (n=4,049), or with unrecorded sex (n=196), maternity care (n=7,873) or for adolescents who died in hospital (n=498). A further 8,942 admissions with abdominal pain were excluded because a likely medical cause was recorded. We analysed the remaining 210,441 admissions (32% of the 662,000 emergency admissions identified) with a stress-related presentation.

**Figure 1:**
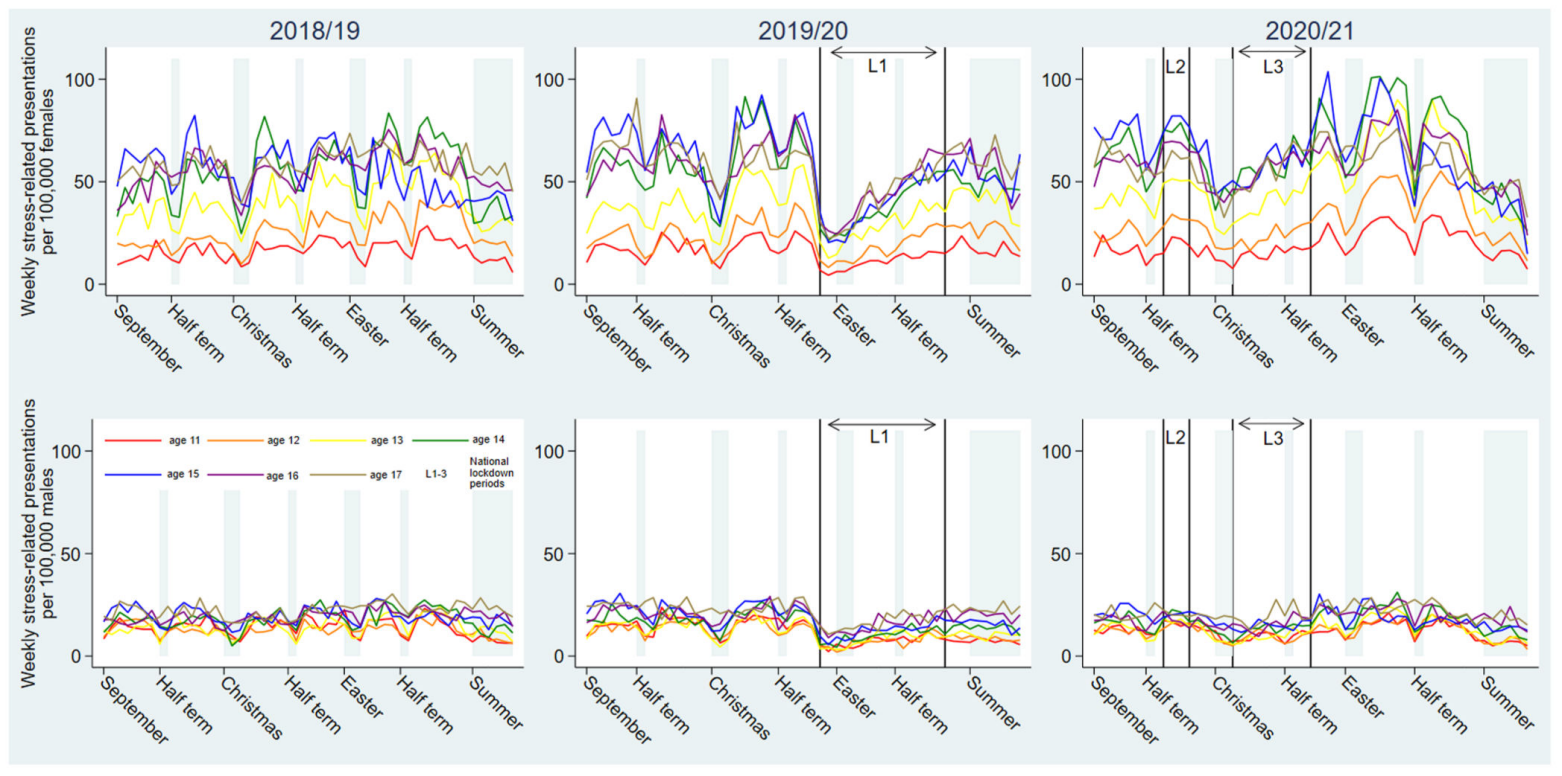
Weekly rates of hospital admissions with stress-related conditions per 100,000 adolescent girls or boys in the academic years of 2018/19, 2019/20 and 2020/21. Figure note: school holiday periods are shown in light grey and the three national lockdown periods (L1-3) are indicated on the x axis. L1; March 20th- July 3rd 2020, L2; November 5th-December 1st 2020, L3; January 6th-March 7th 2021. Schools closed to the majority of pupils on March 20th 2020 with attendance for secondary school aged children remaining very low (<5%) for the remainder of the academic year 2019/20 [4].

Figure 1 shows the weekly rate of emergency admissions in the three academic years examined. The rate of stress-related presentations was highest in girls, particularly those aged 14-15 years (Supplementary Figure 1). During the first lockdown there was a rapid decline in stress-related presentations – including the loss of term-time peaks – that slightly preceded the closure of schools on March 20^th^ 2020. This was followed by a rebound in rates of stress-related presentations and resumption of term-time peaks to historic levels by July 2020. The small number of pandemic holiday periods (Easter and summer half-term 2020) when schools were closed limits scope for formal evaluation of changes in term versus holiday presentations.

Across the three years investigated, mean weekly term-time rates of stress-related presentations were higher than holiday-time rates for girls of all ages and boys aged 11-16 years (Supplementary Figures 1 and 2). Compared to 2018/19, incident rate ratios for weekly rates of stress-related presentations were 17-22% higher in 2020/21 for girls aged 11-15, but were unchanged for boys, and girls aged 16-17 years (Supplementary Figure 3). A total of 43% (90,392) of stress presentations were classified as mental health/behavioural, with the remaining 57% (120,049) pain/somatic. The proportion of stress presentations that were mental health/behavioural increased from 40% in 2018/19 to 46% in 2020/21 (Chi p<0.001).

## Discussion

Our main findings are that following the start of the pandemic, rates of hospital admission with stress-related presentations have increased substantially for girls aged 11-15 years, reflecting higher rates in both term-time and holiday periods. In boys, stress-related admission rates were much lower than for girls and remain at pre-pandemic levels. The observed increase for girls corroborates reports of declining levels of adolescent wellbeing exacerbated by insufficient support, as illustrated by a 94% increase in referrals to Child and Adolescent Mental Health services in 2021 relative to 2019 [1,5].

We found that term-time rates of stress-related admissions were higher than holiday periods for girls and boys aged 11-16 years. This is similar to pre-pandemic data for England [2] and Ireland, where patterns of CAMHS referrals reduced during holiday times [6]. Qualitative research is needed to determine whether the term-time peaks reflect schools as drivers of stress (e.g. through academic pressures or bullying) or if schools increase identification of distressed pupils and facilitate help-seeking.

We also identified a rapid drop in stress-related presentations that preceded the closure of schools on March 20^th^ 2020 [4]. This is similar to that reported more widely, including a 50-65% decline in contacts for paediatric psychiatric and self-harm presentations [1]. This sharp decline most likely reflects reduced healthcare attendance (e.g. due to fear of nosocomial covid infection), rather than reduced need.

As our data on hospitalisations captures only the tip of the iceberg of stress manifestations the true burden will be substantially greater and warrants ongoing action from researchers, schools and clinicians. Stress-related presentations accounted for almost one third of all emergency hospital admissions in 11 to 17 year olds. This indicates a real need for earlier intervention in other settings as well as interdisciplinary support at admission - potentially through mental health liaison service - to address the bio-psycho-social factors that are likely contributing to the need for emergency care. Further research should use linked clinical data to examine referral patterns and service use associated with these stress presentations [7] and determine their suitability for evaluations of mental health interventions, including those delivered through schools.

## Supplementary Material

Supplementary material

## Data Availability

HES data are available on application to the NHS Digital (https://digital.nhs.uk/).

## References

[1] Viner R, Russell S, Saulle R (2022). School Closures During Social Lockdown and Mental Health, Health Behaviors, and Well-being Among Children and Adolescents During the First COVID-19 Wave: A Systematic Review. JAMA Pediatr.

[2] Blackburn R, Ajetunmobi O, Mc Grath-Lone L, Hardelid P, Shafran R, Gilbert R, Wijlaars L (2021). Hospital admissions for stress-related presentations among school-aged adolescents during term time versus holidays in England: Weekly time series and retrospective cross-sectional analysis. BJPsych Open.

[3] Jensen PS, Goldman E, Offord D (2011). Overlooked and underserved: “Action signs” for identifying children with unmet mental health needs. Pediatrics.

[4] DfE Learning during the pandemic: quantifying lost time.

[5] House of Commons Health and Social Care Committee Clearing the backlog caused by the pandemic.

[6] McNicholas F, Kelleher I, Hedderman E (2021). Referral patterns for specialist child and adolescent mental health services in the Republic of Ireland during the COVID-19 pandemic compared with 2019 and 2018. BJPsych Open.

[7] Perera G, Broadbent M, Callard F (2016). Cohort profile of the South London and Maudsley NHS Foundation trust biomedical research centre (SLAM BRC) case register: current status and recent enhancement of an electronic mental health Record-derived data resource. BMJ Open.

