# Supplementary material for: School closures and patterns of hospital admissions with stress-related presentations in secondary school aged adolescents
Source: Br J Psychiatry. Author manuscript; Available in PMC 2022 Nov 1. (PMC7613736; doi:10.1192/bjp.2022.113)
Supplement: Supplementary material [file EMS151335-supplement-Supplementary_material.pdf]

Supplementary Figure 1) Mean weekly rates of stress-related presentations in term and holiday times in 2018/19 and 2020/21 by sex and year of age.

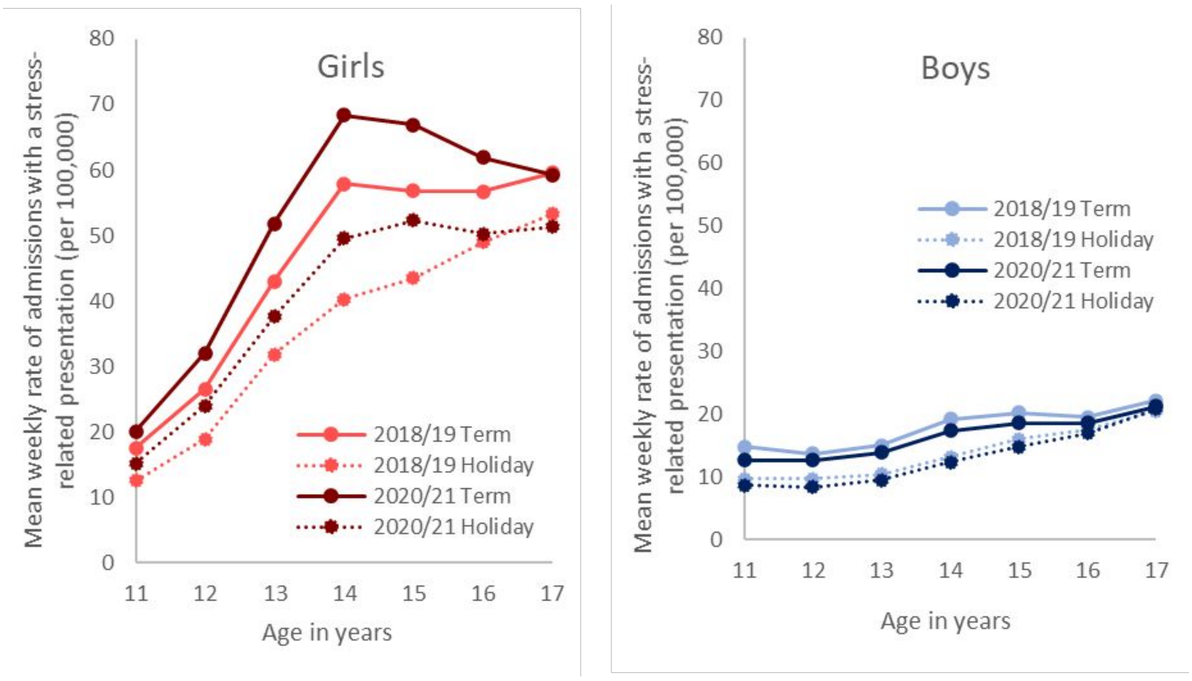

Figure note: data points reflect discreet 1 year age bands. Connecting lines are shown for ease of reading. Data for the year 2019/20 are not shown. Overall mean weekly rates of stress-related admissions were higher in term times than holidays for girls and boys of all ages (t test of weekly counts, $p<0.005$ in all instances), except boys aged 17 years ($p=0.11$).

Supplementary Figures 2 and 3 show age and sex-specific incident rate ratios (IRRs) and associated 95% confidence intervals for the weekly rate of admission with a stress-related presentation in 2018/19-2020/21. **Figure 1** shows IRRs for term-time (versus holiday periods) and **Figure 2** shows IRRs for admissions in 2020/21 (versus 2018/19).

Figure 2

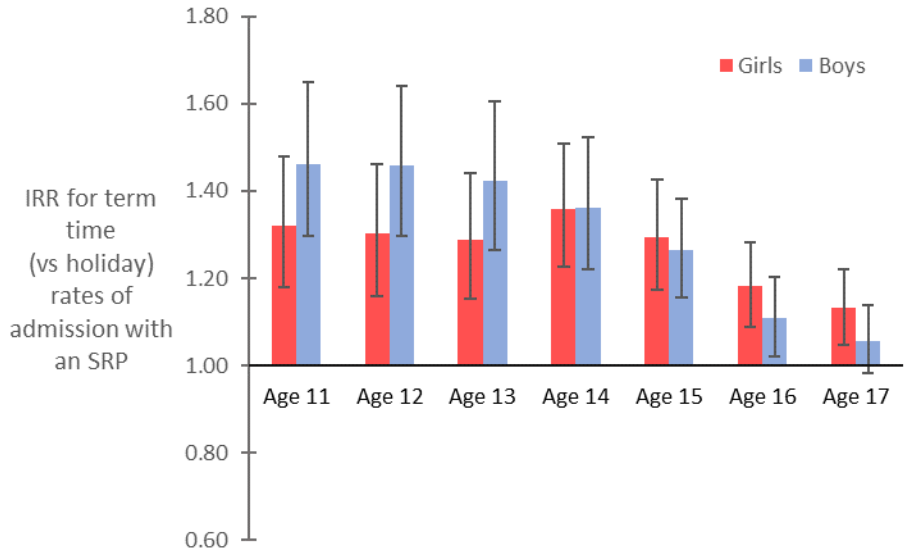

Figure 3

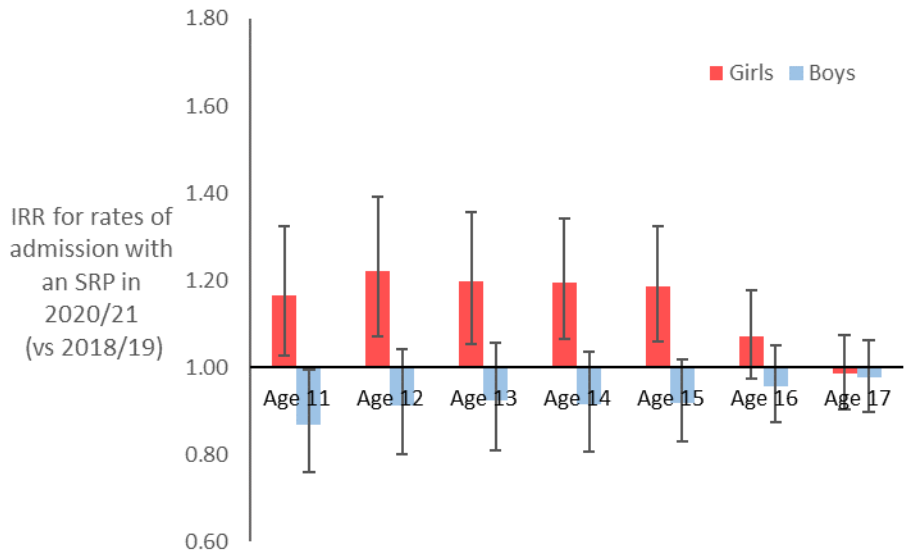

Figure note: IRRs were estimated from age and sex-specific negative binomial regression models for the weekly rate of admission with a stress-related presentation. Models included mid-year population estimates as an offset, term (vs holiday) as the exposure and year of admission as a covariate (3-level factor).

Supplementary Table 1: International Classification of Diseases and Related Health Problems version 10 (ICD-10) code list for stress-related presentations

Category	Group	ICD-code	ICD-10 Description
Pain-related presentations	Abdominal/pelvic pain	R10 [1]	Abdominal and pelvic pain
	Headache	R51	Headache
		G442	Tension-type headache
	Other pain	M54	Panniculitis affecting regions of neck and back
		M626	Muscle strain
		M796	Pain in limb
		R52	Acute pain
Other somatic presentations	Circulatory/respiratory signs	R00	Abnormalities of heart beat
		R03	Abnormal blood pressure reading, without diagnosis
		R05	Cough
		R06	Abnormalities of breathing
		R07	Pain in throat and chest
	Digestive symptoms	R11-14	Nausea and vomiting, Heartburn, Dysphagia, Flatulence and related conditions
		R194	Change in bowel habit
	Skin symptoms	R20-21	Disturbances of skin sensation, rash and other nonspecific skin eruption
		R231	Pallor
		R234	Changes in skin texture
		R238	Other and unspecified skin changes
	Nervous/musculoskeletal symptoms	R25	Abnormal involuntary movements
		R26	Abnormalities of gait and mobility
		R27	Other lack of co-ordination

Category	Group	ICD-code	ICD-10 Description
		R292-294	Abnormal reflex, Abnormal posture, Clicking hip
		R298	Other and unspecified signs and symptoms involving nervous and musculoskeletal systems
	Cognitive symptoms	R400-401	Somnolence, Stupor
		R41-42	Other symptoms and signs involving cognitive functions and awareness, Dizziness and giddiness
Other somatic presentations (continued)	Malaise/Fatigue/Syncope	R53	Malaise and fatigue
		R55	Syncope and collapse
	Other/general symptoms	R44	Other symptoms and signs involving general sensations and perceptions
		R45	Symptoms and signs involving emotional state
		R46	Symptoms and signs involving appearance and behaviour
		R47	Dysphasia and aphasia
		R49	Voice disturbances
		Z563-564	Stressful work schedule, Discord with boss and workmates
		Z711	Person with feared complaint in whom no diagnosis is made
		Z733	Stress, not elsewhere classified
Mental health & behavioural presentations	Anxiety/Depression	F31	Bipolar affective disorder
		F320	Mild depressive episode
		F321	Moderate depressive episode
		F322	Severe depression without psychotic symptoms
		F323	Severe depression with psychotic symptoms
		F328	Other depressive episodes
		F329	Depressive episode, unspecified
		F330	Recurrent depressive disorder, current episode mild

Category	Group	ICD-code	ICD-10 Description
		F331	Recurrent depressive disorder, current episode moderate
		F332	Recurrent depressive disorder, current episode severe without psychotic symptoms
		F333	Recurrent depressive disorder, current episode severe with psychotic symptoms
		F338	Other recurrent depressive disorders
		F339	Recurrent depressive disorder, unspecified
		F341	Dysthymia
		F40	Agoraphobia
		F410	Social phobias
		F411	Generalized anxiety disorder
		F412	Mixed anxiety and depressive disorder
Mental health & behavioural presentations (cont.)	Anxiety/Depression (cont.)	F43	Other mixed anxiety disorder
	Mental health	F20-F29	Schizophrenia, schizotypal and delusional disorders
		F30-F39	Mood disorders
		F40-F49	Neurotic, stress-related and somatoform disorders
		F50-F59	Behavioural syndromes associated with physiological disturbances and physical factors
		F60-F69	Disorders of adult personality and behaviour
		F90-F98	Behavioural and emotional disorders with onset usually occurring in childhood and adolescence
		F99	Unspecified mental disorder
	Obsessive-compulsive / Dissociative / Eating disorders	F42	Obsessive-compulsive disorder
		F44	Dissociative [conversation] disorders
		F45	Somatoform disorders
		F50	Eating disorders
	Sleep disorders	F51	Nonorganic sleep disorders

Category	Group	ICD-code	ICD-10 Description
		G47	Disorders of initiating and maintaining sleep [insomnias]
	Drug / Alcohol abuse	F10-F19	Mental and behavioural disorders due to psychoactive substance use
		F55	Abuse of non-dependence-producing substances
		R780	Finding of alcohol in blood
		R781-R785	Findings of drugs and other substances, not normally found in blood
		T51	Toxic effect of alcohol
		Y15 [2]	Poisoning by drugs, medicaments and biological substances
		Y90 [2, 3]	Evidence of alcohol involvement determined by blood alcohol level
		Y91 [2, 3]	Evidence of alcohol involvement determined by level of intoxication
		Z040 [2, 3]	Blood-alcohol and blood-drug test
		Z502 [2, 3]	Alcohol rehabilitation
		Z503 [2, 3]	Drug rehabilitation
		Z714 [2, 3]	Alcohol abuse counselling and surveillance
		Z715 [2, 3]	Drug abuse counselling and surveillance
		Z721 [2, 3]	Alcohol use
Mental health & behavioural presentations (cont.)	Drug / Alcohol abuse (cont.)	Z722 [2, 3]	Drug use
		Z915 [2, 4]	Personal history of self-harm
	Self-harm / Poisoning / Cutting	T36-50 [3]	Poisoning by drugs, medicaments and biological substances
		Y10-14 [2]	Poisoning [drugs], undetermined intent
		Y16-19 [2]	Poisoning [chemicals], undetermined intent
		S00 [3]	Superficial injury of scalp
		S10 [3]	Superficial injury of neck
		S20 [3]	Superficial injury of thorax
		S30 [3]	Superficial injury of lower back and pelvis

Category	Group	ICD-code	ICD-10 Description
		S40 [3]	Superficial injury of shoulder and upper arm
		S50 [3]	Superficial injury of forearm
		S60 [3]	Superficial injury of wrist and hand
		S70 [3]	Superficial injury of hip and thigh
		S80 [3]	Superficial injury of lower leg
		S90 [3]	Superficial injury of ankle and foot
		X60-63 [2]	Intentional self-poisoning (drugs)
		X64-X69 [2]	Intentional self-harm (self-poisoning)
		X70-X84 2	Intentional self-harm (hanging, drowning, firearm, explosive material, fire, steam, sharp/blunt object, jumping, crashing motor vehicle, other)
		Z642 [2]	Intentional self-poisoning by and exposure to other and unspecified drugs, medicaments and biological substances
		Z915 [2, 4]	Personal history of self-harm

ICD-10 = International Classification of Diseases and Related Health Problems version 10. Supplementary Table 1 outlines the ICD-10 code list that was developed as part of this study to identify a stress-related presentation in Hospital Episode Statistics Admitted Patient Care (HES APC) data based on information recorded in the diagnosis fields. In HES APC up to 20 diagnosis fields can be recorded per admission using ICD-10 codes. An admission was categorised as a stress-related presentation if an ICD-10 code listed in Supplementary Table 1 was recorded in the primary diagnostic position, or in any diagnostic position for some mental health and behavioural presentations, as indicated.[1] Admissions with a primary diagnostic code of R10 (Abdominal and pelvic pain) were not categorised as a stress-related presentation if a medical or surgical cause was indicated by an operation or subsidiary diagnostic code for the same admission (as detailed in Supplementary Table 2).[2] An admission was categorised as a stress-related presentation if this ICD-10 code was recorded in any diagnostic coding position.[3] An admission was categorised as a stress-related presentation if this ICD-10 code was recorded in the primary diagnostic coding position <u>and</u> one of the following self-harm codes were also recorded in another diagnostic position: X60-63 (Intentional self-poisoning

(drugs)), X64-X69 (Intentional self-harm (self-poisoning)), X70-X84 (Intentional self-harm (hanging, drowning, firearm, explosive material, fire, steam, sharp/blunt object, jumping, crashing motor vehicle, other)), Z642 (Intentional self-poisoning by and exposure to other and unspecified drugs, medicaments and biological substances) or Z915 (personal history of self-harm).[4] For admissions that included Z915 (personal history of self-harm), where the diagnostic codes reflected more than one category (e.g., drug/alcohol abuse and self-harm presentations), the presentation was classified as Self-harm/ Poisoning / Cutting only, such that the groups were mutually exclusive.

Supplementary Table 2) Diagnostic and operation codes indicating a medical or surgical cause for presentations of abdominal pain

	**Code**	**Description**
Diagnosis	N832	Other and unspecified ovarian cysts
	A099	Gastroenteritis and colitis of unspecified origin
	K529	Noninfective gastroenteritis and colitis, unspecified
	E282	Polycystic ovarian syndrome
	N390	Urinary tract infection, site not specified
	K589	Irritable bowel syndrome without diarrhoea
Operation	Y752	Laparoscopic approach to the abdominal cavity not elsewhere classified
	H013	Emergency excision of normal appendix
	H029	Unspecified excision of appendix
	H012	Emergency excision of normal appendix not elsewhere classified
	H019	Unspecified emergency excision of appendix
	H021	Interval appendectomy
	H023	Prophylactic appendectomy NEC
	H024	Incidental appendectomy
	H028	Other specified excision of appendix
	H011	Emergency excision of abnormal appendix and drainage HFQ

In Hospital Episode Statistics Admitted Patient Care data, up to 20 diagnosis fields and 24 operation fields can be recorded per admission using standardised coding systems (International Classification of Diseases and Related Health Problems version 10 (ICD-10) codes for diagnoses and Office of Population Censuses and Surveys version 4 (OPCS-4) for operations). Admissions with a primary diagnostic code of R10 (Abdominal and pelvic pain) were not categorised as a stress-related presentation if a medical or surgical cause was indicated by a subsidiary diagnostic code or any operation code listed in Supplementary Material 4 recorded for the same admission.
